# Supplementary material for: Modeling glioblastoma heterogeneity as a dynamic network of cell states
Source: Mol Syst Biol. 2021 Sep 16;17(9):e10105. doi: 10.15252/msb.202010105 (PMC8444284; doi:10.15252/msb.202010105)
Supplement: Supplementary file 5 — Source Data for Figure 3 [file MSB-17-e10105-s001.zip › Figure3A_sourcedata/GSEA_3065/nosigdb_state6.GseaPreranked.1622718443993/RICHARDS_NOWAKOWSKI_SCIENCE_2017_MGE_RG2_UPREG.html]

Details for gene set RICHARDS\_NOWAKOWSKI\_SCIENCE\_2017\_MGE\_RG2\_UPREG[GSEA]

|  || Dataset | state6 |
| Phenotype | NoPhenotypeAvailable |
| Upregulated in class | na\_pos |
| GeneSet | RICHARDS\_NOWAKOWSKI\_SCIENCE\_2017\_MGE\_RG2\_UPREG |
| Enrichment Score (ES) | 0.36736375 |
| Normalized Enrichment Score (NES) | 1.872867 |
| Nominal p-value | 0.0 |
| FDR q-value | 0.004095326 |
| FWER p-Value | 0.05 |
Table: GSEA Results Summary

  

Fig 1: Enrichment plot: RICHARDS\_NOWAKOWSKI\_SCIENCE\_2017\_MGE\_RG2\_UPREG      
 Profile of the Running ES Score & Positions of GeneSet Members on the Rank Ordered List

  

| PROBE | GENE SYMBOL | GENE\_TITLE | RANK IN GENE LIST | RANK METRIC SCORE | RUNNING ES | CORE ENRICHMENT || 1 | RPLP1 |  |  | 6 | 0.329 | 0.0068 | Yes |
| 2 | EEF1A1 |  |  | 9 | 0.325 | 0.0140 | Yes |
| 3 | RPLP0 |  |  | 10 | 0.312 | 0.0211 | Yes |
| 4 | RPS27 |  |  | 11 | 0.307 | 0.0282 | Yes |
| 5 | RPS19 |  |  | 14 | 0.295 | 0.0347 | Yes |
| 6 | PABPC1 |  |  | 15 | 0.292 | 0.0414 | Yes |
| 7 | RPL37A |  |  | 16 | 0.290 | 0.0480 | Yes |
| 8 | RPL13 |  |  | 21 | 0.286 | 0.0541 | Yes |
| 9 | RPL3 |  |  | 22 | 0.285 | 0.0606 | Yes |
| 10 | RPS28 |  |  | 24 | 0.283 | 0.0670 | Yes |
| 11 | RPLP2 |  |  | 31 | 0.271 | 0.0724 | Yes |
| 12 | CCND2 |  |  | 32 | 0.269 | 0.0786 | Yes |
| 13 | RPL10 |  |  | 33 | 0.265 | 0.0847 | Yes |
| 14 | RPL18 |  |  | 36 | 0.256 | 0.0903 | Yes |
| 15 | RPL6 |  |  | 38 | 0.254 | 0.0960 | Yes |
| 16 | RPL18A |  |  | 39 | 0.252 | 0.1017 | Yes |
| 17 | RPL41 |  |  | 42 | 0.249 | 0.1072 | Yes |
| 18 | HES6 |  |  | 44 | 0.248 | 0.1128 | Yes |
| 19 | RPL29 |  |  | 47 | 0.247 | 0.1182 | Yes |
| 20 | RPS8 |  |  | 51 | 0.245 | 0.1234 | Yes |
| 21 | RPS13 |  |  | 55 | 0.243 | 0.1286 | Yes |
| 22 | RPS4X |  |  | 58 | 0.238 | 0.1339 | Yes |
| 23 | RPS3 |  |  | 59 | 0.238 | 0.1393 | Yes |
| 24 | RPL4 |  |  | 60 | 0.237 | 0.1447 | Yes |
| 25 | RPL32 |  |  | 69 | 0.231 | 0.1490 | Yes |
| 26 | RPS14 |  |  | 72 | 0.229 | 0.1540 | Yes |
| 27 | JUND |  |  | 75 | 0.226 | 0.1590 | Yes |
| 28 | RPL7A |  |  | 76 | 0.226 | 0.1641 | Yes |
| 29 | PEA15 |  |  | 79 | 0.225 | 0.1691 | Yes |
| 30 | RPS3A |  |  | 80 | 0.224 | 0.1742 | Yes |
| 31 | RPL39 |  |  | 82 | 0.223 | 0.1792 | Yes |
| 32 | RPS25 |  |  | 84 | 0.223 | 0.1842 | Yes |
| 33 | NACA |  |  | 86 | 0.221 | 0.1891 | Yes |
| 34 | RPL5 |  |  | 90 | 0.218 | 0.1937 | Yes |
| 35 | PTMA |  |  | 94 | 0.216 | 0.1983 | Yes |
| 36 | SERF2 |  |  | 97 | 0.215 | 0.2030 | Yes |
| 37 | RPL15 |  |  | 98 | 0.215 | 0.2079 | Yes |
| 38 | SET |  |  | 100 | 0.213 | 0.2127 | Yes |
| 39 | GLCCI1 |  |  | 101 | 0.213 | 0.2176 | Yes |
| 40 | RPS16 |  |  | 103 | 0.212 | 0.2223 | Yes |
| 41 | HNRNPA1 |  |  | 108 | 0.209 | 0.2266 | Yes |
| 42 | MZT2B |  |  | 113 | 0.206 | 0.2308 | Yes |
| 43 | TP53 |  |  | 116 | 0.205 | 0.2353 | Yes |
| 44 | RPL12 |  |  | 121 | 0.203 | 0.2395 | Yes |
| 45 | OLIG2 |  |  | 126 | 0.201 | 0.2436 | Yes |
| 46 | RPL31 |  |  | 135 | 0.198 | 0.2472 | Yes |
| 47 | RPS27A |  |  | 136 | 0.198 | 0.2517 | Yes |
| 48 | SOX9 |  |  | 148 | 0.193 | 0.2548 | Yes |
| 49 | NAA50 |  |  | 152 | 0.190 | 0.2588 | Yes |
| 50 | TMSB4X |  |  | 162 | 0.187 | 0.2620 | Yes |
| 51 | ZFAND6 |  |  | 163 | 0.187 | 0.2663 | Yes |
| 52 | RPL36 |  |  | 166 | 0.187 | 0.2703 | Yes |
| 53 | RPL27A |  |  | 183 | 0.182 | 0.2726 | Yes |
| 54 | RPS18 |  |  | 185 | 0.181 | 0.2766 | Yes |
| 55 | RPS15 |  |  | 193 | 0.178 | 0.2798 | Yes |
| 56 | BTF3 |  |  | 194 | 0.178 | 0.2839 | Yes |
| 57 | GLUL |  |  | 199 | 0.177 | 0.2874 | Yes |
| 58 | PARP1 |  |  | 206 | 0.175 | 0.2907 | Yes |
| 59 | UQCRH |  |  | 208 | 0.174 | 0.2946 | Yes |
| 60 | RPL8 |  |  | 215 | 0.170 | 0.2978 | Yes |
| 61 | RPSA |  |  | 219 | 0.169 | 0.3013 | Yes |
| 62 | RPL10A |  |  | 233 | 0.166 | 0.3035 | Yes |
| 63 | AIF1L |  |  | 237 | 0.165 | 0.3069 | Yes |
| 64 | WWTR1 |  |  | 241 | 0.164 | 0.3103 | Yes |
| 65 | RPL23A |  |  | 246 | 0.163 | 0.3136 | Yes |
| 66 | TMSB15A |  |  | 253 | 0.161 | 0.3165 | Yes |
| 67 | RPL13A |  |  | 255 | 0.161 | 0.3201 | Yes |
| 68 | ZFP36L1 |  |  | 266 | 0.159 | 0.3225 | Yes |
| 69 | RPL9 |  |  | 290 | 0.153 | 0.3233 | Yes |
| 70 | NME4 |  |  | 309 | 0.149 | 0.3246 | Yes |
| 71 | GNG5 |  |  | 326 | 0.147 | 0.3260 | Yes |
| 72 | HNRNPC |  |  | 331 | 0.147 | 0.3289 | Yes |
| 73 | EIF3E |  |  | 334 | 0.146 | 0.3320 | Yes |
| 74 | CORO1C |  |  | 345 | 0.145 | 0.3341 | Yes |
| 75 | LSM2 |  |  | 356 | 0.142 | 0.3362 | Yes |
| 76 | FOXN3 |  |  | 369 | 0.140 | 0.3380 | Yes |
| 77 | RPL27 |  |  | 382 | 0.139 | 0.3397 | Yes |
| 78 | YBX1 |  |  | 384 | 0.139 | 0.3427 | Yes |
| 79 | RFC4 |  |  | 397 | 0.136 | 0.3444 | Yes |
| 80 | NUTF2 |  |  | 410 | 0.134 | 0.3461 | Yes |
| 81 | TCF7L1 |  |  | 427 | 0.131 | 0.3472 | Yes |
| 82 | YBX3 |  |  | 432 | 0.131 | 0.3497 | Yes |
| 83 | CHIC2 |  |  | 433 | 0.130 | 0.3527 | Yes |
| 84 | RPL23 |  |  | 457 | 0.127 | 0.3528 | Yes |
| 85 | TFDP2 |  |  | 507 | 0.122 | 0.3498 | Yes |
| 86 | ZC3HAV1 |  |  | 552 | 0.118 | 0.3472 | Yes |
| 87 | RPL21 |  |  | 554 | 0.118 | 0.3498 | Yes |
| 88 | HDAC9 |  |  | 574 | 0.116 | 0.3501 | Yes |
| 89 | HSDL2 |  |  | 606 | 0.113 | 0.3490 | Yes |
| 90 | RAD23A |  |  | 622 | 0.112 | 0.3498 | Yes |
| 91 | RPS6 |  |  | 647 | 0.110 | 0.3495 | Yes |
| 92 | HNRNPUL1 |  |  | 657 | 0.110 | 0.3509 | Yes |
| 93 | HMGN2 |  |  | 679 | 0.108 | 0.3508 | Yes |
| 94 | EEF1D |  |  | 682 | 0.107 | 0.3531 | Yes |
| 95 | TMEM263 |  |  | 690 | 0.107 | 0.3547 | Yes |
| 96 | LITAF |  |  | 692 | 0.106 | 0.3570 | Yes |
| 97 | APBB2 |  |  | 693 | 0.106 | 0.3594 | Yes |
| 98 | RPL35 |  |  | 756 | 0.102 | 0.3543 | Yes |
| 99 | TLE3 |  |  | 765 | 0.101 | 0.3557 | Yes |
| 100 | HIST1H1C |  |  | 768 | 0.101 | 0.3577 | Yes |
| 101 | SRRM1 |  |  | 776 | 0.100 | 0.3592 | Yes |
| 102 | CHCHD2 |  |  | 796 | 0.099 | 0.3592 | Yes |
| 103 | SNRPD2 |  |  | 805 | 0.098 | 0.3605 | Yes |
| 104 | CDK6 |  |  | 807 | 0.097 | 0.3626 | Yes |
| 105 | QKI |  |  | 820 | 0.097 | 0.3633 | Yes |
| 106 | MIS12 |  |  | 825 | 0.096 | 0.3651 | Yes |
| 107 | ANAPC11 |  |  | 841 | 0.095 | 0.3655 | Yes |
| 108 | PTPRN2 |  |  | 854 | 0.094 | 0.3662 | Yes |
| 109 | SOX2 |  |  | 863 | 0.093 | 0.3674 | Yes |
| 110 | PTPN13 |  |  | 983 | 0.087 | 0.3551 | No |
| 111 | CNBP |  |  | 997 | 0.086 | 0.3555 | No |
| 112 | TOX3 |  |  | 999 | 0.086 | 0.3574 | No |
| 113 | H2AFY |  |  | 1011 | 0.085 | 0.3580 | No |
| 114 | PRDX2 |  |  | 1061 | 0.082 | 0.3540 | No |
| 115 | NUCKS1 |  |  | 1066 | 0.082 | 0.3554 | No |
| 116 | RPL7 |  |  | 1076 | 0.082 | 0.3562 | No |
| 117 | NR1D2 |  |  | 1123 | 0.080 | 0.3525 | No |
| 118 | BBX |  |  | 1139 | 0.078 | 0.3525 | No |
| 119 | GLO1 |  |  | 1161 | 0.077 | 0.3518 | No |
| 120 | PSD3 |  |  | 1180 | 0.076 | 0.3514 | No |
| 121 | MRPL45 |  |  | 1192 | 0.076 | 0.3518 | No |
| 122 | RFTN2 |  |  | 1196 | 0.075 | 0.3532 | No |
| 123 | CREB5 |  |  | 1231 | 0.074 | 0.3508 | No |
| 124 | TMEM106C |  |  | 1286 | 0.072 | 0.3460 | No |
| 125 | ZKSCAN1 |  |  | 1303 | 0.071 | 0.3457 | No |
| 126 | TROVE2 |  |  | 1316 | 0.071 | 0.3459 | No |
| 127 | CKB |  |  | 1359 | 0.069 | 0.3424 | No |
| 128 | MBIP |  |  | 1390 | 0.068 | 0.3404 | No |
| 129 | ALDH6A1 |  |  | 1436 | 0.066 | 0.3365 | No |
| 130 | CTNNB1 |  |  | 1461 | 0.066 | 0.3352 | No |
| 131 | ABAT |  |  | 1484 | 0.065 | 0.3340 | No |
| 132 | CDK4 |  |  | 1490 | 0.064 | 0.3349 | No |
| 133 | DEK |  |  | 1535 | 0.063 | 0.3311 | No |
| 134 | TIMELESS |  |  | 1584 | 0.061 | 0.3267 | No |
| 135 | RDX |  |  | 1603 | 0.060 | 0.3259 | No |
| 136 | GLTP |  |  | 1672 | 0.058 | 0.3191 | No |
| 137 | USP1 |  |  | 1712 | 0.056 | 0.3158 | No |
| 138 | RHOA |  |  | 1836 | 0.052 | 0.3023 | No |
| 139 | ZBTB20 |  |  | 1892 | 0.051 | 0.2968 | No |
| 140 | RPS10 |  |  | 1934 | 0.050 | 0.2931 | No |
| 141 | BAG4 |  |  | 1944 | 0.049 | 0.2931 | No |
| 142 | CHD7 |  |  | 1953 | 0.049 | 0.2933 | No |
| 143 | ANXA5 |  |  | 1978 | 0.048 | 0.2915 | No |
| 144 | ANXA7 |  |  | 1981 | 0.048 | 0.2924 | No |
| 145 | PRPS2 |  |  | 2001 | 0.048 | 0.2912 | No |
| 146 | C12orf29 |  |  | 2056 | 0.046 | 0.2858 | No |
| 147 | TUBA1B |  |  | 2087 | 0.045 | 0.2833 | No |
| 148 | COA4 |  |  | 2117 | 0.044 | 0.2808 | No |
| 149 | CDO1 |  |  | 2145 | 0.043 | 0.2786 | No |
| 150 | NAP1L1 |  |  | 2171 | 0.043 | 0.2765 | No |
| 151 | BLM |  |  | 2242 | 0.040 | 0.2691 | No |
| 152 | TLK1 |  |  | 2244 | 0.040 | 0.2699 | No |
| 153 | CBX5 |  |  | 2269 | 0.039 | 0.2679 | No |
| 154 | DKC1 |  |  | 2283 | 0.039 | 0.2673 | No |
| 155 | FAM111B |  |  | 2365 | 0.037 | 0.2584 | No |
| 156 | DERA |  |  | 2397 | 0.036 | 0.2555 | No |
| 157 | CCT3 |  |  | 2419 | 0.036 | 0.2538 | No |
| 158 | WDR76 |  |  | 2452 | 0.035 | 0.2508 | No |
| 159 | GSTP1 |  |  | 2453 | 0.035 | 0.2516 | No |
| 160 | MCM6 |  |  | 2484 | 0.034 | 0.2488 | No |
| 161 | BAZ1A |  |  | 2495 | 0.033 | 0.2484 | No |
| 162 | DPH3 |  |  | 2536 | 0.032 | 0.2443 | No |
| 163 | ANP32E |  |  | 2570 | 0.032 | 0.2411 | No |
| 164 | MRPL24 |  |  | 2576 | 0.031 | 0.2412 | No |
| 165 | NOTCH3 |  |  | 2595 | 0.031 | 0.2398 | No |
| 166 | TIMM10 |  |  | 2632 | 0.030 | 0.2362 | No |
| 167 | CDC7 |  |  | 2650 | 0.030 | 0.2348 | No |
| 168 | TRIM16 |  |  | 2720 | 0.028 | 0.2272 | No |
| 169 | SFRP1 |  |  | 2812 | 0.026 | 0.2169 | No |
| 170 | E2F7 |  |  | 2818 | 0.026 | 0.2169 | No |
| 171 | PHF10 |  |  | 2879 | 0.025 | 0.2103 | No |
| 172 | TMPO |  |  | 2978 | 0.022 | 0.1991 | No |
| 173 | CKS1B |  |  | 2985 | 0.022 | 0.1989 | No |
| 174 | SRSF3 |  |  | 3000 | 0.022 | 0.1977 | No |
| 175 | TMA16 |  |  | 3018 | 0.022 | 0.1962 | No |
| 176 | UTP20 |  |  | 3053 | 0.021 | 0.1926 | No |
| 177 | LARP1B |  |  | 3089 | 0.020 | 0.1889 | No |
| 178 | SRSF6 |  |  | 3114 | 0.020 | 0.1865 | No |
| 179 | ARL4A |  |  | 3127 | 0.019 | 0.1855 | No |
| 180 | AXL |  |  | 3293 | 0.016 | 0.1661 | No |
| 181 | YAP1 |  |  | 3295 | 0.016 | 0.1663 | No |
| 182 | EIF3M |  |  | 3355 | 0.014 | 0.1596 | No |
| 183 | HNRNPD |  |  | 3359 | 0.014 | 0.1596 | No |
| 184 | NEDD1 |  |  | 3369 | 0.014 | 0.1588 | No |
| 185 | RBBP8 |  |  | 3390 | 0.013 | 0.1567 | No |
| 186 | LSM6 |  |  | 3391 | 0.013 | 0.1570 | No |
| 187 | IGSF11 |  |  | 3446 | 0.012 | 0.1508 | No |
| 188 | POLA1 |  |  | 3456 | 0.012 | 0.1500 | No |
| 189 | NOTCH1 |  |  | 3459 | 0.012 | 0.1501 | No |
| 190 | CDC5L |  |  | 3466 | 0.012 | 0.1496 | No |
| 191 | SALL1 |  |  | 3467 | 0.012 | 0.1499 | No |
| 192 | PDLIM3 |  |  | 3503 | 0.011 | 0.1459 | No |
| 193 | PRIM1 |  |  | 3532 | 0.011 | 0.1428 | No |
| 194 | HSPB1 |  |  | 3587 | 0.010 | 0.1366 | No |
| 195 | DUT |  |  | 3596 | 0.009 | 0.1359 | No |
| 196 | RFC2 |  |  | 3618 | 0.009 | 0.1336 | No |
| 197 | GPATCH4 |  |  | 3641 | 0.008 | 0.1311 | No |
| 198 | HNRNPF |  |  | 3709 | 0.007 | 0.1233 | No |
| 199 | FRMD5 |  |  | 3722 | 0.006 | 0.1220 | No |
| 200 | ALYREF |  |  | 3755 | 0.006 | 0.1183 | No |
| 201 | PRTFDC1 |  |  | 3762 | 0.006 | 0.1177 | No |
| 202 | FBL |  |  | 3777 | 0.005 | 0.1161 | No |
| 203 | SNRPC |  |  | 3859 | 0.004 | 0.1065 | No |
| 204 | PCDH17 |  |  | 3912 | 0.002 | 0.1003 | No |
| 205 | PPIA |  |  | 3964 | 0.001 | 0.0943 | No |
| 206 | LRR1 |  |  | 3977 | 0.001 | 0.0929 | No |
| 207 | TSPAN6 |  |  | 3995 | 0.001 | 0.0908 | No |
| 208 | TPM2 |  |  | 4007 | 0.001 | 0.0895 | No |
| 209 | DIS3L |  |  | 4062 | -0.000 | 0.0831 | No |
| 210 | DAXX |  |  | 4136 | -0.002 | 0.0744 | No |
| 211 | NCL |  |  | 4158 | -0.002 | 0.0720 | No |
| 212 | SLC25A5 |  |  | 4174 | -0.003 | 0.0702 | No |
| 213 | LIN54 |  |  | 4287 | -0.005 | 0.0569 | No |
| 214 | MDK |  |  | 4311 | -0.005 | 0.0543 | No |
| 215 | HAUS5 |  |  | 4351 | -0.006 | 0.0498 | No |
| 216 | G2E3 |  |  | 4365 | -0.006 | 0.0484 | No |
| 217 | CCT5 |  |  | 4370 | -0.006 | 0.0480 | No |
| 218 | SNRPB |  |  | 4387 | -0.007 | 0.0463 | No |
| 219 | SLC35F1 |  |  | 4421 | -0.007 | 0.0425 | No |
| 220 | CRKL |  |  | 4422 | -0.007 | 0.0426 | No |
| 221 | MCM7 |  |  | 4435 | -0.008 | 0.0414 | No |
| 222 | ILDR2 |  |  | 4444 | -0.008 | 0.0406 | No |
| 223 | DDAH1 |  |  | 4446 | -0.008 | 0.0407 | No |
| 224 | SNRPD1 |  |  | 4477 | -0.008 | 0.0373 | No |
| 225 | TRMT10B |  |  | 4501 | -0.009 | 0.0347 | No |
| 226 | FOS |  |  | 4536 | -0.010 | 0.0309 | No |
| 227 | HAUS1 |  |  | 4556 | -0.010 | 0.0288 | No |
| 228 | LDHB |  |  | 4572 | -0.010 | 0.0273 | No |
| 229 | RFC5 |  |  | 4594 | -0.011 | 0.0250 | No |
| 230 | MCM10 |  |  | 4638 | -0.011 | 0.0201 | No |
| 231 | SEMA5A |  |  | 4642 | -0.012 | 0.0200 | No |
| 232 | UHRF1 |  |  | 4792 | -0.014 | 0.0025 | No |
| 233 | SMAD5 |  |  | 4873 | -0.016 | -0.0067 | No |
| 234 | CHCHD3 |  |  | 4885 | -0.016 | -0.0076 | No |
| 235 | SKP2 |  |  | 4907 | -0.017 | -0.0098 | No |
| 236 | GNL3 |  |  | 4977 | -0.018 | -0.0176 | No |
| 237 | LIMD1 |  |  | 4986 | -0.018 | -0.0181 | No |
| 238 | NUP62 |  |  | 5002 | -0.018 | -0.0195 | No |
| 239 | IQGAP2 |  |  | 5055 | -0.019 | -0.0253 | No |
| 240 | ZFP36L2 |  |  | 5069 | -0.020 | -0.0264 | No |
| 241 | C5orf30 |  |  | 5074 | -0.020 | -0.0264 | No |
| 242 | MTA3 |  |  | 5116 | -0.021 | -0.0308 | No |
| 243 | C19orf48 |  |  | 5117 | -0.021 | -0.0304 | No |
| 244 | NUP153 |  |  | 5222 | -0.023 | -0.0423 | No |
| 245 | NSRP1 |  |  | 5248 | -0.023 | -0.0448 | No |
| 246 | C9orf3 |  |  | 5280 | -0.024 | -0.0479 | No |
| 247 | FKBP10 |  |  | 5319 | -0.025 | -0.0519 | No |
| 248 | CCDC138 |  |  | 5357 | -0.025 | -0.0557 | No |
| 249 | STK39 |  |  | 5381 | -0.026 | -0.0579 | No |
| 250 | NPM1 |  |  | 5412 | -0.027 | -0.0609 | No |
| 251 | LYRM2 |  |  | 5442 | -0.027 | -0.0637 | No |
| 252 | HNRNPA3 |  |  | 5463 | -0.028 | -0.0655 | No |
| 253 | SF3A3 |  |  | 5467 | -0.028 | -0.0652 | No |
| 254 | NME1 |  |  | 5479 | -0.028 | -0.0659 | No |
| 255 | INPP5F |  |  | 5488 | -0.028 | -0.0662 | No |
| 256 | DCAF16 |  |  | 5497 | -0.028 | -0.0665 | No |
| 257 | DYNC2H1 |  |  | 5499 | -0.028 | -0.0660 | No |
| 258 | PBX3 |  |  | 5501 | -0.028 | -0.0654 | No |
| 259 | CTPS1 |  |  | 5522 | -0.029 | -0.0672 | No |
| 260 | SOAT1 |  |  | 5529 | -0.029 | -0.0672 | No |
| 261 | SSB |  |  | 5596 | -0.030 | -0.0744 | No |
| 262 | TRIM26 |  |  | 5602 | -0.031 | -0.0743 | No |
| 263 | TRIM9 |  |  | 5619 | -0.031 | -0.0755 | No |
| 264 | WDHD1 |  |  | 5630 | -0.031 | -0.0760 | No |
| 265 | PRDX3 |  |  | 5634 | -0.031 | -0.0756 | No |
| 266 | PRMT5 |  |  | 5662 | -0.032 | -0.0781 | No |
| 267 | MNS1 |  |  | 5869 | -0.035 | -0.1020 | No |
| 268 | EIF2S1 |  |  | 5897 | -0.036 | -0.1044 | No |
| 269 | CEP83 |  |  | 5921 | -0.036 | -0.1063 | No |
| 270 | JAM2 |  |  | 5934 | -0.037 | -0.1069 | No |
| 271 | CEBPZ |  |  | 5976 | -0.038 | -0.1109 | No |
| 272 | SNX4 |  |  | 6032 | -0.039 | -0.1166 | No |
| 273 | EPHB2 |  |  | 6035 | -0.039 | -0.1160 | No |
| 274 | CUTA |  |  | 6038 | -0.039 | -0.1153 | No |
| 275 | RFC3 |  |  | 6078 | -0.040 | -0.1191 | No |
| 276 | H2AFZ |  |  | 6079 | -0.040 | -0.1181 | No |
| 277 | FAM111A |  |  | 6097 | -0.040 | -0.1193 | No |
| 278 | AHI1 |  |  | 6098 | -0.040 | -0.1183 | No |
| 279 | WRN |  |  | 6102 | -0.040 | -0.1178 | No |
| 280 | SRI |  |  | 6182 | -0.042 | -0.1263 | No |
| 281 | SFRP2 |  |  | 6191 | -0.042 | -0.1262 | No |
| 282 | POLI |  |  | 6201 | -0.043 | -0.1263 | No |
| 283 | DTL |  |  | 6209 | -0.043 | -0.1262 | No |
| 284 | CCDC59 |  |  | 6252 | -0.044 | -0.1302 | No |
| 285 | RIC3 |  |  | 6255 | -0.044 | -0.1295 | No |
| 286 | NUP43 |  |  | 6257 | -0.044 | -0.1286 | No |
| 287 | SEPT10 |  |  | 6275 | -0.044 | -0.1296 | No |
| 288 | BARD1 |  |  | 6284 | -0.044 | -0.1296 | No |
| 289 | CEP152 |  |  | 6298 | -0.044 | -0.1301 | No |
| 290 | RANBP1 |  |  | 6309 | -0.045 | -0.1303 | No |
| 291 | PARD3 |  |  | 6399 | -0.047 | -0.1398 | No |
| 292 | GINS2 |  |  | 6461 | -0.049 | -0.1460 | No |
| 293 | FANCI |  |  | 6473 | -0.049 | -0.1462 | No |
| 294 | RPA2 |  |  | 6501 | -0.050 | -0.1483 | No |
| 295 | BMS1 |  |  | 6512 | -0.050 | -0.1483 | No |
| 296 | EGR1 |  |  | 6519 | -0.050 | -0.1479 | No |
| 297 | SRSF10 |  |  | 6530 | -0.050 | -0.1480 | No |
| 298 | GAB1 |  |  | 6546 | -0.051 | -0.1486 | No |
| 299 | CD99 |  |  | 6572 | -0.052 | -0.1504 | No |
| 300 | PDLIM5 |  |  | 6585 | -0.052 | -0.1506 | No |
| 301 | SRSF7 |  |  | 6593 | -0.053 | -0.1502 | No |
| 302 | VRK1 |  |  | 6626 | -0.053 | -0.1528 | No |
| 303 | SCAF11 |  |  | 6653 | -0.054 | -0.1547 | No |
| 304 | SRSF1 |  |  | 6655 | -0.054 | -0.1536 | No |
| 305 | PHGDH |  |  | 6659 | -0.054 | -0.1527 | No |
| 306 | HAUS6 |  |  | 6688 | -0.055 | -0.1548 | No |
| 307 | MKI67 |  |  | 6750 | -0.057 | -0.1608 | No |
| 308 | MCM5 |  |  | 6765 | -0.058 | -0.1611 | No |
| 309 | PPIL4 |  |  | 6810 | -0.059 | -0.1650 | No |
| 310 | CDC37 |  |  | 6861 | -0.061 | -0.1696 | No |
| 311 | BCAN |  |  | 6866 | -0.061 | -0.1687 | No |
| 312 | NUDT5 |  |  | 6878 | -0.061 | -0.1686 | No |
| 313 | FANCD2 |  |  | 6903 | -0.062 | -0.1701 | No |
| 314 | CCNE2 |  |  | 6916 | -0.062 | -0.1701 | No |
| 315 | PRC1 |  |  | 6918 | -0.062 | -0.1688 | No |
| 316 | EXOSC9 |  |  | 6932 | -0.063 | -0.1689 | No |
| 317 | MTCL1 |  |  | 6949 | -0.063 | -0.1694 | No |
| 318 | BIRC5 |  |  | 7024 | -0.066 | -0.1767 | No |
| 319 | KIF22 |  |  | 7098 | -0.068 | -0.1839 | No |
| 320 | G3BP1 |  |  | 7109 | -0.068 | -0.1835 | No |
| 321 | AASS |  |  | 7119 | -0.068 | -0.1830 | No |
| 322 | APEX1 |  |  | 7171 | -0.070 | -0.1875 | No |
| 323 | FAM161A |  |  | 7176 | -0.070 | -0.1864 | No |
| 324 | HMGB1 |  |  | 7186 | -0.071 | -0.1859 | No |
| 325 | PA2G4 |  |  | 7192 | -0.071 | -0.1848 | No |
| 326 | FEN1 |  |  | 7218 | -0.071 | -0.1862 | No |
| 327 | RAN |  |  | 7221 | -0.072 | -0.1848 | No |
| 328 | RAD51AP1 |  |  | 7236 | -0.072 | -0.1848 | No |
| 329 | UNG |  |  | 7249 | -0.073 | -0.1846 | No |
| 330 | MIS18BP1 |  |  | 7253 | -0.073 | -0.1833 | No |
| 331 | RSL1D1 |  |  | 7349 | -0.076 | -0.1929 | No |
| 332 | LIPG |  |  | 7356 | -0.076 | -0.1919 | No |
| 333 | TMEM18 |  |  | 7357 | -0.076 | -0.1901 | No |
| 334 | SRRM2 |  |  | 7389 | -0.077 | -0.1921 | No |
| 335 | FUT8 |  |  | 7393 | -0.078 | -0.1907 | No |
| 336 | HSPD1 |  |  | 7433 | -0.079 | -0.1935 | No |
| 337 | CENPC |  |  | 7474 | -0.080 | -0.1965 | No |
| 338 | RRM1 |  |  | 7491 | -0.081 | -0.1965 | No |
| 339 | C1orf35 |  |  | 7492 | -0.081 | -0.1947 | No |
| 340 | HAT1 |  |  | 7538 | -0.083 | -0.1981 | No |
| 341 | ENO1 |  |  | 7559 | -0.084 | -0.1986 | No |
| 342 | FDPS |  |  | 7587 | -0.085 | -0.1999 | No |
| 343 | MCM3 |  |  | 7597 | -0.086 | -0.1990 | No |
| 344 | PIKFYVE |  |  | 7612 | -0.087 | -0.1987 | No |
| 345 | ZEB1 |  |  | 7621 | -0.087 | -0.1977 | No |
| 346 | DBF4 |  |  | 7639 | -0.088 | -0.1977 | No |
| 347 | LDLR |  |  | 7648 | -0.088 | -0.1966 | No |
| 348 | GMNN |  |  | 7651 | -0.088 | -0.1948 | No |
| 349 | MMAB |  |  | 7655 | -0.089 | -0.1932 | No |
| 350 | EXOSC8 |  |  | 7671 | -0.089 | -0.1929 | No |
| 351 | PKM |  |  | 7732 | -0.093 | -0.1980 | No |
| 352 | TPI1 |  |  | 7757 | -0.093 | -0.1987 | No |
| 353 | BRCA2 |  |  | 7772 | -0.094 | -0.1982 | No |
| 354 | ESCO2 |  |  | 7778 | -0.094 | -0.1967 | No |
| 355 | TMEM123 |  |  | 7786 | -0.095 | -0.1954 | No |
| 356 | STAG2 |  |  | 7830 | -0.097 | -0.1983 | No |
| 357 | ATP1B3 |  |  | 7835 | -0.098 | -0.1965 | No |
| 358 | CHEK1 |  |  | 7852 | -0.099 | -0.1962 | No |
| 359 | MCM4 |  |  | 7900 | -0.101 | -0.1995 | No |
| 360 | SCD |  |  | 7908 | -0.102 | -0.1980 | No |
| 361 | ZNF121 |  |  | 7914 | -0.102 | -0.1963 | No |
| 362 | HNRNPA2B1 |  |  | 7941 | -0.103 | -0.1970 | No |
| 363 | ITGB3BP |  |  | 7959 | -0.104 | -0.1966 | No |
| 364 | CHAF1A |  |  | 7967 | -0.105 | -0.1951 | No |
| 365 | RAD18 |  |  | 7983 | -0.106 | -0.1944 | No |
| 366 | LIG1 |  |  | 7987 | -0.106 | -0.1924 | No |
| 367 | ING5 |  |  | 8008 | -0.108 | -0.1923 | No |
| 368 | CDK2 |  |  | 8018 | -0.108 | -0.1909 | No |
| 369 | POLD3 |  |  | 8033 | -0.109 | -0.1901 | No |
| 370 | SMC1A |  |  | 8047 | -0.110 | -0.1891 | No |
| 371 | TMEM97 |  |  | 8049 | -0.110 | -0.1867 | No |
| 372 | WEE1 |  |  | 8054 | -0.111 | -0.1847 | No |
| 373 | PCNA |  |  | 8061 | -0.111 | -0.1829 | No |
| 374 | PAM |  |  | 8068 | -0.111 | -0.1810 | No |
| 375 | RBL1 |  |  | 8069 | -0.111 | -0.1785 | No |
| 376 | CKS2 |  |  | 8077 | -0.112 | -0.1768 | No |
| 377 | PRKDC |  |  | 8083 | -0.113 | -0.1748 | No |
| 378 | NCAPD3 |  |  | 8089 | -0.113 | -0.1728 | No |
| 379 | MCM2 |  |  | 8099 | -0.114 | -0.1713 | No |
| 380 | MSI2 |  |  | 8115 | -0.115 | -0.1704 | No |
| 381 | CNTLN |  |  | 8150 | -0.117 | -0.1718 | No |
| 382 | PON2 |  |  | 8177 | -0.120 | -0.1722 | No |
| 383 | CYR61 |  |  | 8178 | -0.120 | -0.1694 | No |
| 384 | RFC1 |  |  | 8205 | -0.122 | -0.1698 | No |
| 385 | MMS22L |  |  | 8222 | -0.124 | -0.1688 | No |
| 386 | KIF15 |  |  | 8228 | -0.124 | -0.1666 | No |
| 387 | EGFR |  |  | 8251 | -0.126 | -0.1664 | No |
| 388 | LDHA |  |  | 8261 | -0.127 | -0.1645 | No |
| 389 | TYMS |  |  | 8287 | -0.131 | -0.1645 | No |
| 390 | CCDC14 |  |  | 8299 | -0.132 | -0.1628 | No |
| 391 | BRIP1 |  |  | 8314 | -0.134 | -0.1614 | No |
| 392 | NASP |  |  | 8318 | -0.134 | -0.1587 | No |
| 393 | CDCA7 |  |  | 8337 | -0.137 | -0.1577 | No |
| 394 | CLIC1 |  |  | 8343 | -0.137 | -0.1552 | No |
| 395 | CDK1 |  |  | 8351 | -0.139 | -0.1528 | No |
| 396 | ATAD2 |  |  | 8359 | -0.141 | -0.1505 | No |
| 397 | FAM114A1 |  |  | 8364 | -0.141 | -0.1477 | No |
| 398 | HMGB2 |  |  | 8388 | -0.145 | -0.1471 | No |
| 399 | KNTC1 |  |  | 8395 | -0.146 | -0.1445 | No |
| 400 | CENPU |  |  | 8403 | -0.147 | -0.1420 | No |
| 401 | SLC16A1 |  |  | 8415 | -0.148 | -0.1399 | No |
| 402 | MAD2L1 |  |  | 8427 | -0.150 | -0.1378 | No |
| 403 | CLSPN |  |  | 8440 | -0.151 | -0.1358 | No |
| 404 | BSG |  |  | 8443 | -0.151 | -0.1326 | No |
| 405 | SPECC1 |  |  | 8467 | -0.154 | -0.1318 | No |
| 406 | ORC6 |  |  | 8482 | -0.157 | -0.1299 | No |
| 407 | PDPN |  |  | 8486 | -0.158 | -0.1266 | No |
| 408 | ARHGAP5 |  |  | 8494 | -0.160 | -0.1238 | No |
| 409 | NUSAP1 |  |  | 8523 | -0.167 | -0.1233 | No |
| 410 | PNN |  |  | 8524 | -0.167 | -0.1195 | No |
| 411 | PABPN1 |  |  | 8553 | -0.173 | -0.1189 | No |
| 412 | MSH6 |  |  | 8568 | -0.177 | -0.1165 | No |
| 413 | DNMT1 |  |  | 8582 | -0.184 | -0.1139 | No |
| 414 | DHFR |  |  | 8590 | -0.186 | -0.1104 | No |
| 415 | RCN1 |  |  | 8602 | -0.191 | -0.1074 | No |
| 416 | ALG8 |  |  | 8603 | -0.192 | -0.1030 | No |
| 417 | NOP56 |  |  | 8619 | -0.199 | -0.1002 | No |
| 418 | PDIA3 |  |  | 8647 | -0.212 | -0.0986 | No |
| 419 | PAICS |  |  | 8653 | -0.214 | -0.0943 | No |
| 420 | SMC2 |  |  | 8658 | -0.216 | -0.0899 | No |
| 421 | BRCA1 |  |  | 8659 | -0.217 | -0.0849 | No |
| 422 | PRDX4 |  |  | 8660 | -0.217 | -0.0799 | No |
| 423 | CENPK |  |  | 8666 | -0.220 | -0.0755 | No |
| 424 | NES |  |  | 8677 | -0.227 | -0.0715 | No |
| 425 | FAT1 |  |  | 8679 | -0.228 | -0.0664 | No |
| 426 | CENPF |  |  | 8703 | -0.240 | -0.0636 | No |
| 427 | HELLS |  |  | 8720 | -0.260 | -0.0596 | No |
| 428 | TMX1 |  |  | 8738 | -0.275 | -0.0553 | No |
| 429 | SMC4 |  |  | 8764 | -0.307 | -0.0513 | No |
| 430 | CD63 |  |  | 8776 | -0.335 | -0.0450 | No |
| 431 | EMP2 |  |  | 8781 | -0.350 | -0.0374 | No |
| 432 | HSPA5 |  |  | 8785 | -0.369 | -0.0293 | No |
| 433 | TNC |  |  | 8787 | -0.389 | -0.0206 | No |
| 434 | PDIA6 |  |  | 8792 | -0.448 | -0.0108 | No |
| 435 | HSP90B1 |  |  | 8794 | -0.487 | 0.0002 | No |
Table: GSEA details [plain text format]

  

Fig 2: RICHARDS\_NOWAKOWSKI\_SCIENCE\_2017\_MGE\_RG2\_UPREG: Random ES distribution      
 Gene set null distribution of ES for **RICHARDS\_NOWAKOWSKI\_SCIENCE\_2017\_MGE\_RG2\_UPREG**
